# Association between primary Spanish language and quality of intrapartum care among Latina women: a secondary analysis of the Listening to Mothers in California survey

**DOI:** 10.1186/s12884-023-05526-4

**Published:** 2023-03-28

**Authors:** Jessica J. Valdez, Andrea V. Jackson, Cassondra Marshall

**Affiliations:** 1 School of Public Health, University of California, Berkeley, Berkeley, CA USA; 2 University of California, San Francisco, School of Medicine, 513 Parnassus Ave, S-245, San Francisco, CA 94143 USA; 3 Department of Obstetrics, Gynecology, and Reproductive Sciences, University of California, San Francisco, CA USA

**Keywords:** Language barriers, Patient-centered, Intrapartum, Maternal care, Quality of care, Limited English proficiency, Latina, Spanish-speaking

## Abstract

**Background:**

Language barriers play significant roles in quality of healthcare. Limited studies have examined the relationships between Spanish language and quality of intrapartum care. The objective was to determine the association between primary Spanish language and quality of intrapartum care so as to further inform best practices for non-English speaking patients in the labor and delivery setting.

**Methods:**

We used the 2016 Listening to Mothers in California survey data, which included a statewide representative sample of women who gave birth in hospitals. Our analytical sample included 1202 Latina women. Multivariable logistic regression was used to examine the association between primary language (monolingual English vs. monolingual Spanish vs. bilingual Spanish/English) and perceived discrimination due to language, perceived pressure for medical interventions, and mistreatment during labor, adjusting for maternal sociodemographics and other maternal and neonatal factors.

**Results:**

Over one-third of the study population spoke English (35.6%), less than one-third spoke Spanish (29.1%), and greater than one-third spoke bilingual Spanish/English (35.3%). Overall, 5.4% of Latina women perceived discrimination due to language spoken, 23.1% perceived pressure for any medical intervention, and 10.1% experienced either form of mistreatment. Compared to English-speakers, Spanish-speakers were significantly more likely to report discrimination due to language (aOR 4.36; 95% CI 1.15–16.59), but were significantly less likely to experience pressure for certain medical interventions (labor induction or cesarean delivery) during labor (aOR 0.34; 95% CI 0.15–0.79 for induction; aOR 0.44; 95% CI 0.18–0.97 for cesarean delivery). Bilingual Spanish/English-speakers also significantly reported discrimination due to language to a lesser extent than monolingual Spanish-speakers (aOR 3.37; 95% CI 1.12–10.13). Any form of Spanish language (monolingual or bilingual) was not significantly associated with mistreatment.

**Conclusions:**

Spanish language may contribute to experiences of discrimination during intrapartum care among Latina women. Future research is needed to explore perceptions of pressure, discrimination and mistreatment, among patients with limited English proficiency.

## Background

Language barriers in all fields of medicine prohibit patient autonomy and dampen quality of care. Language barriers are associated with poor quality of care related to access issues [1], untimely preventive care [2], medical complications or adverse events [3, 4], as well as poor healthcare quality such as lower patient satisfaction and patient-centered care [5]. From 2009 to 2013, approximately 25 million people in the United States (US) were considered limited English proficient (LEP), with numbers continuing to rise, and the majority of those LEP persons speaking monolingual Spanish [6]. Given this data, culturally-sensitive and linguistically fluent care that is focused on Latinx health is essential. This is particularly important with respect to maternal healthcare. Recent data shows that Latina women have the second highest fertility rate compared to other racial/ethnic groups, indicating more pregnant patients requiring high-quality maternal care with cultural and language support [7]. Intrapartum or birthing care, is an important area of focus given that the majority of births occur in hospitals [8], and hospital birthing care is viewed as an integral part of the maternal patient experience. Patient language is a major predictor of quality of care thus, one important aspect of high-quality maternal care among Spanish-speaking patients involves focusing on eliminating disparities associated with language by first identifying such disparities.

For context, there are many disparities and inequities present in maternal health outcomes and maternal care in the US, including care that occurs before birth (antepartum), during birth (intrapartum), and after birth (postpartum) [9]. For example, there are stark racial/ethnic disparities in the maternal mortality rate for Black and Indigenous women, which is 3–4 times and 2 times higher, respectively, than that of their white counterparts [10, 11]. Nationally, Latina women experience both elevated severe maternal morbidity and in-hospital deaths relative to white women [12]. Other disparities, like higher cesarean delivery rates at US-Mexico border counties, as well as other severe maternal morbidities based on location exist among this ethnic group [13, 14].

Quality of maternal care is important to consider given that high quality care is essential to the preservation of health rights and the implementation of health equity [15]. The World Health Organization (WHO) defines quality of care as “the extent to which health care services provided to individuals and patient populations improve desired health outcomes,” and includes seven domains of quality of care such as, safety, timeliness, equity, integration, efficiency, efficacy, and people-centeredness [16]. There are numerous studies examining clinical maternal health outcomes such as maternal mortality, cesarean delivery rates, etc., however there is a smaller body of literature focusing on other important maternal quality of care indicators, such as patient-centeredness, patient perceptions of care, and patient satisfaction. Evaluating patient-centered quality of care measures is just as important as examining clinical obstetric outcomes, given that they contribute to better overall health outcomes, enhance the therapeutic patient-provider relationship, and lead to improved overall healthcare delivery in the US [17–19]. Notably, one study using the national survey-based Listening to Mothers dataset of 2400 pregnant women consisting of various racial/ethnic groups (white, Black, Latina, or other) in the US found that those who perceived pressure from providers to undergo labor induction or cesarean delivery were significantly more likely to undergo labor induction or cesarean delivery [20]. Additionally, other studies using similar national datasets have shown that women of color are more likely to experience disrespectful care, mistreatment, and discrimination during their birth experience due to their race/ethnicity [21, 22]. A study using a subset of the national Listening to Mothers dataset with pregnant women specific to California (also known as the Listening to Mothers in California survey) showed similar results of perceived pressure of labor induction or cesarean delivery leading to labor induction or cesarean delivery respectively, as in the national dataset [23]. Another study involving the Listening to Mothers in California dataset with 2,318 pregnant women in California found that those who had Medicaid as their primary form of insurance were more likely to experience lack of autonomy and increased pressure by providers for cesarean delivery [24]. The patient-centeredness component of quality of care is an important aspect of maternal health that needs to be improved but has been understudied. Furthermore, available research has not thoroughly considered the role of language barriers and patient-centered quality of maternal care among non-English speaking patients.

Limited studies have investigated maternal health, particularly birthing care, and language. Few quantitative studies have looked at Spanish-speakers and their experiences with prenatal care, with one study of 125 Latina women showing that Spanish-speaking patients tended to experience more communication issues compared to their English-speaking, non-Latina counterparts [25]. Some qualitative studies have also examined patient preferences in communication with their providers during prenatal care among migrant Latina women, with patients regarding the physician’s ability to speak Spanish during a clinic visit as an integral component of patient-centered care [26, 27].

Currently, there is one study that has examined language or LEP status and quality of intrapartum care, specifically with clinical obstetric outcomes, among Asian American & Pacific Islander (AAPI) women [28]. This study of 11,419 AAPI and white participants in Hawaii, found that patients speaking an Asian language were more likely to have increased rates of cesarean deliveries and increased risk of obstetric trauma (defined as perineal tears or lacerations) in vaginal deliveries without instrumentation, compared to their English-speaking counterparts [28]. Overall, the available body of research suggests that speaking a language other than English may be associated with poorer quality of maternal care outcomes.

To our knowledge, there are no studies to date that examine the relationship between speaking Spanish as a primary language and intrapartum patient-centered quality of care outcomes among Latina women. Such inquiry could enable further policy-level discussions of ways to eliminate and prevent poor quality of care among limited English proficient, immigrant birthing communities. Our study used a California statewide representative sample to investigate the occurrence of differences in patient-centered outcomes during labor among Latina women based on their primary language. We hypothesized that speaking Spanish as a primary language increases the likelihood that a participant would experience discrimination due to their language, pressure for medical interventions during labor (epidural administration, labor induction, and cesarean delivery), and mistreatment (verbal and physical mistreatment) during labor.

## Materials & Methods

### Study design & participants

Our study was a secondary analysis of the population-based Listening to Mothers in California (LTM-CA) cross-sectional survey of 2539 participants. Given that the LTM-CA survey recruited presumed women and did not further ask participants about their gender identity, the language to identify participants (i.e. “women, mothers”) used in our study mirrors that of the survey. LTM-CA is a statewide continuation of the national Listening to Mothers (LTM) surveys first established in 2002, all of which assess for maternal experiences and opinions of pregnancy, labor, and childbirth. Participants were recruited systematically using California birth certificates. Birth certificate eligibility criteria included women who: had a singleton birth between September 1, 2016 and December 15, 2016; were 18 years or older; were residents of California, were not incarcerated, incapable of taking the survey, or in a rehabilitation facility; were living in the United States at the time of first survey contact; had a hospital birth; whose babies were part of their household at the time of first survey mailing; and who could complete the survey in English or Spanish [29]. Survey questionnaires were administered from February 22, 2017 to August 15, 2017 in English or Spanish over the phone with a language concordant (English vs. Spanish) interviewer or online using any device. Detailed methodology of LTM-CA is described elsewhere [29–31].

For our study, the data was anonymous. We limited the sample to only Latina women (*n* = 1222) who had complete data on the primary language most often spoken in the home and survey language variables (*n* = 1202). Participants were excluded if they responded to the survey as speaking a language other than English or Spanish, and if they had incomplete data on any of the three outcomes of interest (perceived discrimination due to language, perceived pressure for medical interventions, or mistreatment during labor), (*n* = 1202). Other possible outcomes of interest in the data set include: whether granted autonomy was present around the time of birth, whether participants felt well supported, whether participants experienced good communication during their hospital stay, perceived discrimination relating to type of insurance, and perceived discrimination relating to race or ethnicity. The study was not considered human subjects research by the University of California, Berkeley Institutional Review Board. All methods were carried out in accordance with relevant guidelines and regulations.

## Measures

### Independent variable

The independent variable of interest was primary language. The LTM-CA survey includes two questions regarding language: (1) language most often spoken at home and (2) language of the survey itself. The response options for the first question included “English,” “Spanish,” or “English and Spanish equally.” The response options for the second question included “English” or “Spanish” only. We created a categorical exposure variable to assess primary language as “English” (answered “English” for both questions), “Spanish” (answered “Spanish” for both questions), or “Bilingual” (answered “English and Spanish equally” for language most often spoken in home and took survey in “English” or “Spanish”; answered “English” for language spoken at home and took survey in “Spanish”; or answered “Spanish” for language spoken at home and took survey in “English”). We incorporated the survey language variable into our primary language exposure because it may measure ability to communicate in English in health care settings [31]. We also explored the relationship of primary language categorized as a binary variable (English vs. Spanish) instead of a three-level variable through a sensitivity analysis and found that results were similar; we ultimately used the three-level variable since it offered more information about our sample.

### Dependent variables

The dependent variables were intrapartum-associated quality of care outcomes that included: perceived discrimination due to language, perceived pressure for medical interventions, and experiencing mistreatment from a provider or nurse during labor. Perceived discrimination due to language was defined through the survey question that asked, “During your recent hospital stay when you had your baby how often were you treated unfairly because of the language you spoke?” Response options for this question were scaled ranging from “never,” “sometimes,” “usually,” or “always.” Prior literature that utilized this question in national LTM surveys dichotomized this variable (yes—at least once; no—never) due to skewed distribution [22]. Our study population distribution for this variable was also skewed, therefore we also dichotomized this variable into the same response options.

Perceived pressure for medical interventions was demonstrated with three survey questions that asked participants if they “felt pressure from any health professional to: (1) induce labor, (2) use epidural for pain relief, or (3) have a cesarean section.” The response options were binary (yes or no) for these pressure questions. In addition, a composite categorical pressure variable was created that combined all three individual instances of pressure, and represented pressure for at least one of the three medical interventions during labor. The response option for this composite variable was also binary (yes/no) but did not specify individual medical interventions, with the understanding that there are logistical clinical pathway challenges with the construction of this composite variable. A planned cesarean would not involve labor induction, and the decision to have a planned or unplanned cesarean would likely make having epidural analgesia seem welcome and not pressured. Also, the experience of labor induction may reduce the feeling of pressure for epidural, which may be welcome due to the intensity of contractions stimulated by synthetic oxytocin. For these reasons, the separate analyses by intervention make more sense than the composite analysis. The composite variable was only used in the bivariate analysis for simplicity.

Mistreatment by a healthcare provider or nurse was based off of two survey questions that asked participants if, “a nurse or maternity care provider ever handled [them] roughly” (physical mistreatment), or if, “a nurse or maternity care provider ever used harsh rude or threatening language” (verbal mistreatment). Both mistreatment questions had binary response options (yes/no). In addition, a composite categorical mistreatment variable with both physical and verbal mistreatment components was also created, with a binary (yes/no) response. Similar to the perceived pressure outcome, if a participant reported “yes” to having either of the individual types of mistreatment (physical vs. verbal), then they were categorized as having mistreatment (composite).

### Covariates

Covariates were determined a priori through previous literature to determine variables that could have potentially confounded our relationships of interest [20, 22–24]. The following covariates were included: country of origin, mode of delivery for most recent birth, provider present at birth, gestational age, birthweight, socioeconomic status (education, insurance), and maternal factors (maternal age at birth, parity, and relationship status). Each covariate in the multivariable models was categorized as shown in Table 1, with the exception of maternal age and gestational age, which were continuous variables in the models.

**Table 1:** Demographic characteristics of study participants, California, 2016; *n* = 1202

Characteristics	n (weighted %)
***Demographics***	
Maternal age (years)^a^	
18–24	352 (30.0)
25–29	340 (28.4)
30–34	303 (24.9)
35 +	191 (16.7)
Primary language	
English	406 (35.6)
Bilingual (English & Spanish)	422 (35.3)
Spanish	374 (29.1)
Maternal education	
High school or less	592 (50.5)
Some college	410 (35.2)
College	126 (9.5)
Some graduate school or higher	64 (4.7)
Relationship status	
Married	550 (46.4)
Living with someone	425 (35.8)
Single or divorced/separated	209 (17.8)
Insurance type	
Private	350 (30.4)
Medi-Cal or uninsured	829 (69.6)
Parity	
Primiparous	403 (32.1)
Multiparous	799 (67.9)
Mode of delivery for most recent birth	
Vaginal	858 (69.3)
Cesarean	343 (30.7)
Type of provider present at birth	
Obstetrician	764 (67.3)
Midwife	137 (8.6)
Family medicine or other physician	221 (18.9)
Nurse practitioner or physician’s assistant or other	65 (5.2)
Gestational age at birth^a,b^	
< 37 weeks	82 (7.7)
> 37 weeks	1046 (92.3)
Birth weight	
≤ 2500 g	75 (6.5)
2500–3999 g	988 (84.4)
≥ 4000 g	102 (9.0)

^a^ Covariates used as continuous variables in multivariable model

^b^ Gestational age could not be differentiated into preterm, normal term, or late term due to small sample sizes

### Statistical analysis

We conducted descriptive analyses to examine the prevalence of demographic characteristics, using weighted percentages. Chi-squared tests were used with Rao-Scott adjustments to determine associations between covariates and independent and dependent variables. Crude and adjusted odds ratios between primary language and dependent variables were calculated using logistic regression models. Primary English-speakers were the reference group for all models. Statistical significance was set a priori with an alpha level of 0.05. All analyses were performed using weighted data due to the complexity of the survey sampling frame with RStudio version 4.0.2 [32].

## Results

Table 1 describes various characteristics for the study population with weighted percentages. The majority of the estimated study population consisted of Latina women who had some college education or less, had state-sponsored public insurance (Medi-Cal) or were uninsured, were under the age of 30, were multiparous, and were born in the US. Among our sample, a California population estimate of 29.1% of Latina women primarily spoke Spanish.

Table 2 shows covariates by primary language. English-speaking Latina women were significantly more likely to have been born in the US (56.7%) compared to monolingual Spanish-speaking Latina women (4.5%), whereas monolingual Spanish-speaking Latina women were significantly more likely to have been born in a foreign country (64.5%) compared to their English-speaking counterparts (5.8%, *p* < 0.001). English-speaking Latina women were significantly more likely to have some graduate school education (56.8%) compared to monolingual Spanish-speaking Latina women (14.2%), who were significantly more likely to have completed a high school level education (43.1% Spanish-speakers vs. 25.5% English-speakers, *p* < 0.001). A greater proportion of bilingual English and Spanish speakers had some college education (42.1%) than monolingual Spanish-speakers (13.5%), but this proportion was slightly less than English-speakers (44.3%, *p* < 0.001). Of those who had private insurance, a population estimate of 49.1% were English-speaking Latina women, 39.9% were bilingual English and Spanish-speakers, and 10.9% were monolingual Spanish-speakers. Monolingual Spanish-speaking Latina women comprised the majority of those who had public insurance or were uninsured (36.8%, *p* < 0.001). English-speaking Latina women were significantly more likely to have an obstetrician (39.6%) or a midwife (34.8%) as their primary birth provider compared to monolingual Spanish-speakers (26.4% and 30.7%, respectively), whereas monolingual Spanish-speakers were significantly more likely to have a family medicine doctor or other doctor (35.4%), or nurse practitioner, physician’s assistant, or other provider as their primary birth provider (40.0%), compared to English-speakers (25.2% and 23.2%, respectively, *p* < 0.01).

**Table 2:** Covariates by primary language of study participants, California, 2016; *n* = 1202

Characteristics	Primary language *n* = 1202 n (weighted %)			
	English *n* = 406 (35.6%)	Bilingual (English & Spanish equally) *n* = 422 (35.3%)	Spanish *n* = 374 (29.1%)	*p* value^a^
Maternal age (years)^b^				
18–24	122 (36.1)	161 (46.8)	69 (17.1)	< 0.001
25–29	129 (39.9)	118 (34.8)	93 (25.2)	
30–34	105 (37.6)	88 (28.6)	110 (33.8)	
≥ 35	41 (22.3)	51 (26.3)	99 (51.3)	
Country of birth				
U.S	372 (56.7)	255 (38.8)	31 (4.5)	< 0.001
Other country	31 (5.8)	157 (29.8)	333 (64.5)	
Maternal education				
High school or less	137 (25.5)	180 (31.5)	275 (43.1)	< 0.001
Some college	177 (44.3)	174 (42.1)	59 (13.5)	
College	55 (47.2)	47 (35.5)	24 (17.4)	
Some graduate school or higher	34 (56.8)	20 (29.0)	10 (14.2)	
Relationship status				
Married	179 (34.7)	196 (35.5)	175 (29.8)	0.84
Living with someone	143 (36.0)	144 (33.8)	138 (30.2)	
Single or divorced/separated	77 (37.2)	74 (36.6)	58 (26.2)	
Insurance type				
Private	162 (49.1)	146 (39.9)	42 (10.9)	< 0.001
Medi-Cal or uninsured	235 (29.5)	271 (33.7)	323 (36.8)	
Parity				
Primiparous	161 (41.1)	161 (39.7)	81 (19.2)	< 0.001
Multiparous	245 (33.0)	261 (33.2)	293 (33.8)	
Mode of delivery for most recent birth				
Vaginal	290 (36.0)	300 (35.1)	268 (28.9)	0.94
Cesarean	116 (34.9)	121 (35.4)	106 (29.7)	
Type of provider present at birth				
Obstetrician	290 (39.6)	260 (34.0)	214 (26.4)	< 0.01
Midwife	45 (34.8)	48 (34.5)	44 (30.7)	
Family medicine or other physician	51 (25.2)	86 (39.4)	84 (35.4)	
Nurse practitioner or physician’s assistant or other	15 (23.2)	23 (37.2)	27 (40.0)	
Gestational age at birth^b,c^				
< 37 weeks	32 (41.8)	24 (30.6)	26 (27.6)	0.42
> 37 weeks	344 (34.5)	371 (35.7)	331 (29.8)	
Birth weight				
≤ 2500 g	23 (33.4)	24 (33.9)	27 (32.7)	0.67
2500–3999 g	343 (36.5)	343 (35.0)	301 (28.5)	
≥ 4000 g	28 (30.0)	42 (40.1)	32 (29.9)	

^a^ *P* values refer to estimated F test from Rao-Scott corrections to χ^2^ test due to complex sampling design, except where noted

^b^ Covariates used as continuous variables in multivariable model

^c^ Gestational age could not be differentiated into preterm, normal term, or late term due to small sample sizes

Table 3 shows covariates by patient-centered quality of care outcomes in the intrapartum period–perceived discrimination (5.4% prevalence), perceived pressure for medical interventions during labor (23.1% prevalence), and experiences of mistreatment during labor (10.1% prevalence).

**Table 3:** Covariates by primary outcomes, California, 2016; *n* = 1202

Characteristics	Perceived discrimination due to language *n* = 1196 n (weighted %)			Perceived pressure for any medical interventions^a^ *n* = 1184 n (weighted %)			Experienced any form of mistreatment^b^ *n* = 1194 n (weighted %)		
	Yes *n* = 71 (5.4%)	No *n* = 1125 (94.6%)	*p* value^c^	Yes *n* = 266 (23.1%)	No *n* = 918 (76.9%)	*p* value^c^	Yes *n* = 121 (10.1%)	No *n* = 1073 (89.9%)	*p* value^c^
Maternal age (years)^d^									
18–24	24 (5.5)	327 (94.6)	0.36	84 (24.9)	264 (75.1)	0.56	32 (8.2)	318 (91.8)	0.2
25–29	21 (5.9)	317 (94.1)		76 (23.7)	259 (76.3)		39 (12.1)	299 (87.9)	
30–34	11 (3.5)	291 (96.5)		61 (21.0)	236 (79)		25 (8.5)	277 (91.5)	
≥ 35	14 (6.9)	175 (93.1)		39 (20.2)	150 (79.8)		23 (12.5)	165 (87.5)	
Country of birth									
U.S	23 (3.3)	633 (96.7)	< 0.001	175 (27)	473 (73)	< 0.001	61 (9.2)	592 (90.8)	0.19
Other country	46 (8.4)	471 (91.6)		85 (17)	428 (83)		58 (11.6)	460 (88.4)	
Maternal education									
High school or less	47 (7.1)	541 (92.9)	0.01^f^	124 (22.4)	456 (77.6)	0.88	56 (9.2)	532 (90.8)	0.61
Some college	12 (2.8)	397 (97.2)		94 (23.5)	311 (76.5)		43 (10.8)	366 (89.2)	
College	7 (5.1)	118 (94.9)		28 (22.7)	97 (77.3)		15 (12.6)	110 (87.4)	
Some graduate school or higher	3 (4.1)	61 (95.9)		18 (26.9)	46 (73.1)		5 (8.0)	59 (92.0)	
Relationship status									
Married	25 (4.0)	524 (96.0)	< 0.01	128 (24.0)	415 (76.0)	0.81	54 (9.6)	494 (90.4)	0.78
Living with someone	24 (5.1)	399 (94.9)		88 (22.2)	329 (77.8)		41 (10.2)	382 (89.8)	
Single or divorced/separated	22 (9.9)	184 (90.1)		46 (22.4)	161 (77.6)		25 (11.4)	181 (88.6)	
Insurance type									
Private	8 (2.1)	341 (97.9)	< 0.001	87 (25.0)	260 (75.0)	0.36	39 (11.0)	310 (89.0)	0.51
Medi-Cal or uninsured	62 (6.8)	763 (93.2)		175 (22.4)	639 (77.6)		79 (9.7)	744 (90.3)	
Parity									
Primiparous	26 (6.3)	376 (93.7)	0.32	109 (28.4)	290 (71.6)	< 0.01	44 (11.4)	356 (88.6)	0.32
Multiparous	45 (4.9)	749 (95.1)		157 (20.5)	628 (79.5)		77 (9.5)	717 (90.5)	
Mode of delivery for most recent birth									
Vaginal	44 (4.6)	809 (95.4)	0.07	175 (20.9)	674 (79.1)	< 0.05	81 (9.5)	774 (90.5)	0.29
Cesarean	27 (7.1)	316 (92.9)		90 (27.8)	244 (72.2)		40 (11.5)	299 (88.5)	
Type of provider present at birth									
Obstetrician	36 (4.3)	727 (95.7)	0.14^f^	179 (24.1)	575 (75.9)	< 0.05	78 (10.0)	683 (90.0)	0.96
Midwife	9 (6.5)	128 (93.5)		17 (11.5)	119 (88.5)		13 (10.6)	124 (89.4)	
Family medicine or other physician	14 (6.1)	205 (93.9)		47 (22.9)	168 (77.1)		21 (10.2)	198 (89.8)	
Nurse practitioner or physician’s assistant or other	7 (10.2)	56 (89.8)		17 (24.9)	48 (75.1)		6 (8.0)	58 (92.0)	
Gestational age at birth^d,e^									
< 37 weeks	5 (5.6)	77 (94.4)	0.81^f^	16 (21.7)	63 (78.3)	0.87	4 (4.9)	78 (95.1)	0.12
> 37 weeks	60 (5.2)	981 (94.8)		228 (22.6)	806 (77.4)		107 (10.3)	933 (89.7)	
Birth weight									
≤ 2500 g	6 (7.4)	69 (92.6)	0.64^f^	17 (22.8)	57 (77.2)	0.54	7 (9.8)	67 (90.2)	0.69
2500–3999 g	55 (5.0)	928 (95.0)		223 (23.6)	752 (76.4)		98 (9.9)	885 (90.1)	
≥ 4000 g	5 (3.8)	97 (96.2)		20 (18.7)	82 (81.3)		13 (12.7)	88 (87.3)	

^a^ Constructed, composite variable of perceived pressure during labor for epidural, labor induction, or cesarean delivery

^b^ Constructed, composite variable of mistreatment experienced during labor including rough handling or rude, harsh, or threatening language by a nurse or provider

^c^ *P* values refer to estimated F test from Rao-Scott corrections to χ^2^ test due to complex sampling design, except where noted

^d^ Covariates used as continuous variables in multivariable model

^e^ Gestational age could not be differentiated into preterm, normal term, or late term due to small sample sizes

^f^ Fischer's exact test performed using unweighted data due to small sample size of covariates by outcomes

### Perceived discrimination due to language

Significantly more foreign-born Latina women (8.4%) perceived discrimination due to language than US-born Latina women (3.3%, *p* < 0.001). Latina women who completed a high school education (7.1% vs. 2.8% some college, 5.1% college, and 4.1% some grad school or higher, *p* < 0.05) were single or divorced (9.9% vs. 5.1% living with someone and 4.0% married, *p* < 0.01), or had public insurance or were uninsured (6.8% vs. 2.1% private insurance, *p* < 0.001), were significantly more likely to perceive discrimination due to language. Parity, mode of delivery, birth provider, maternal age, gestational age, and birth weight were not significantly associated with perceived discrimination.

### Perceived pressure for medical interventions 

Significantly more US-born Latina women (27.0%) were found to experience pressure for any medical intervention during labor than foreign-born Latina women (17.0%, *p* < 0.001). Primiparous Latina women (28.4%) were significantly more likely to experience pressure for medical interventions than multiparous Latina women (20.5%, *p* < 0.01). Similarly, Latina women who underwent cesarean deliveries (27.8%) were significantly more likely to perceive pressure for medical interventions, compared to Latina women who delivered vaginally (20.9%, *p* < 0.05). Latina women who had midwives as their birth providers (11.5%) experienced significantly less pressure than Latina women who had any other birth providers (24.1% for obstetricians, 22.9% for family medicine physicians or other physicians whom the participants were not sure of or didn’t know the type of specialty, 24.9% for nurse practitioners or physician assistants, *p* < 0.05). Education, relationship status, insurance type, maternal age, gestational age, and birth weight were not significantly associated with the perceived pressure for medical interventions on bivariate analysis.

### Mistreatment (composite)

There were no significant associations between any of the covariates and the composite mistreatment outcome.

Table 4 shows the logistic regression analysis of unadjusted (crude) and adjusted associations between primary language and perceived discrimination due to language during labor. Monolingual Spanish speakers and bilingual Spanish/English speakers were significantly more likely to perceive discrimination due to language on both crude (OR_monolingual_ 5.47, 95% CI: 2.55–11.74; OR_bilingual_ 2.59, 95% CI: 1.15–5.83) and adjusted models. The adjusted odds ratio decreased slightly with monolingual Spanish speakers (aOR_monolingual_ 4.36; 95% CI: 1.15–16.59), while it increased slightly with bilingual Spanish/English-speakers (aOR_bilingual_ 3.37; 95% CI: 1.12–10.13). However, the effect of perceived discrimination was stronger with monolingual Spanish-speakers overall, in both models.

**Table 4:** Multivariable model for perceived language discrimination, California, 2016

Primary language exposure	Perceived discrimination due to language *n* = 1196^a^	
	Crude OR (95% CI)	aOR (95% CI)^b^
English	*Reference*	*Reference*
Bilingual (English & Spanish)	2.59 (1.15–5.83)*	3.37 (1.12–10.13)*
Spanish	5.47 (2.55–11.74)**	4.36 (1.15–16.59)*

*Abbreviations*: *aOR* adjusted odds ratio, *CI* confidence interval, *OR* odds ratio

^a^ Sample sizes for adjusted models were marginally lower due to missing covariate data

^b^ Regression models were adjusted for country of origin, mode of delivery for most recent birth, provider present at birth, gestational age, birthweight, education, insurance, maternal age at birth, parity, and relationship status

^*^p ≤ 0.05

^**^p ≤ 0.001

Table 5 shows the logistic regression analyses of unadjusted and adjusted associations between primary language and the individual perceived pressure outcomes (epidural, labor induction, and cesarean delivery). Results show that Latina women who primarily spoke monolingual Spanish perceived significantly less pressure for individual pressure outcomes of perceived pressure for labor induction (aOR 0.34, 95% CI: 0.15–0.79) and cesarean delivery as well (aOR 0.41, 95% CI: 0.18–0.97). Spanish-speaking participants perceived significantly less pressure for epidural administration on crude analysis (OR 0.53, 95% CI: 0.31–0.93), however this significance disappeared in the adjusted model that controlled for covariates (aOR 0.63, 95% CI: 0.29–1.37).

**Table 5:** Multivariable model for perceived pressure for medical interventions, California, 2016

Primary language exposure	Perceived pressure for epidural *n* = 1189^a^		Perceived pressure for labor induction *n* = 1189^a^		Perceived pressure for cesarean delivery *n* = 1185^a^	
	Crude OR (95% CI)	aOR (95% CI)^b^	Crude OR (95% CI)	aOR (95% CI)^b^	Crude OR (95% CI)	aOR (95% CI)^b^
English	*Reference*	*Reference*	*Reference*	*Reference*	*Reference*	*Reference*
Bilingual (English & Spanish)	1.25 (0.80–1.94)	1.31 (0.77–2.24)	0.72 (0.47–1.08)	0.91 (0.56–1.47)	1.04 (0.67–1.61)	1.03 (0.58–1.83)
Spanish	0.53 (0.31–0.93)*	0.63 (0.29–1.37)	0.29 (0.17–0.51)***	0.34 (0.15–0.79)*	0.44 (0.25–0.76)**	0.41 (0.18–0.97)*

*Abbreviations*: *aOR* adjusted odds ratio, *CI* confidence interval, *OR* odds ratio

^a^ Sample sizes for adjusted models were marginally lower due to missing covariate data

^b^ Regression models were adjusted for country of origin, mode of delivery for most recent birth, provider present at birth, gestational age, birthweight, education, insurance, maternal age at birth, parity, and relationship status

^*^p ≤ 0.05

^**^p ≤ 0.01

^***^p ≤ 0.001

Table 6 shows the logistic regression analyses of unadjusted and adjusted associations between primary language and the composite measure of experiencing mistreatment during labor, as well as disaggregated experiences of mistreatment (either experiencing physical mistreatment or verbal mistreatment). Primary Spanish language was not found to be significantly associated with any of these three outcomes in crude (OR_composite mistreatment_ 1.10, 95% CI: 0.67–1.79) or adjusted logistic regression models (aOR_composite mistreatment_ 0.90, 95% CI: 0.43–1.89).

**Table 6:** Multivariable model for mistreatment during labor, California, 2016

Primary language exposure	Experienced mistreatment by a provider or nurse (composite) *n* = 1194^a^		Experienced physical mistreatment by a provider or nurse *n* = 1195^a^		Experienced verbal mistreatment by a provider or nurse *n* = 1195^a^	
	Crude OR (95% CI)	aOR (95% CI)^b^	Crude OR (95% CI)	aOR (95% CI)^b^	Crude OR (95% CI)	aOR (95% CI)^b^
English	*Reference*	*Reference*	*Reference*	*Reference*	*Reference*	*Reference*
Bilingual (English & Spanish)	1.24 (0.78–1.98)	1.25 (0.72–2.16)	1.07 (0.62–1.84)	1.23 (0.64–2.36)	1.14 (0.66–1.99)	1.08 (0.55–2.15)
Spanish	1.10 (0.67–1.79)	0.90 (0.43–1.89)	0.75 (0.41–1.37)	0.83 (0.35–1.95)	1.04 (0.58–1.86)	0.81 (0.32–2.07)

*Abbreviations*: *aOR* adjusted odds ratio, *CI* confidence interval, *OR* odds ratio

^a^ Sample sizes for adjusted models were marginally lower due to missing covariate data

^b^ Regression models were adjusted for country of origin, mode of delivery for most recent birth, provider present at birth, gestational age, birthweight, education, insurance, maternal age at birth, parity, and relationship status

## Discussion

The objective of this study was to determine the association between Spanish language and quality of maternal care via three measures—perceived language discrimination, perceived pressure by a provider, and mistreatment. In this California population of 1,202 Latina women who had hospital births, we found that participants who spoke any Spanish (either monolingual Spanish or bilingual Spanish/English) were more likely to experience discrimination due to their spoken language during their most recent hospital birth compared to monolingual English-speaking participants, despite experiencing less pressure for any medical intervention during labor, particularly pressure for labor induction and pressure for cesarean delivery. We found no significant association between language and mistreatment.

Language barriers and LEP have been linked to poor quality of care and specifically, decreased patient-centered care. Language disparities have been described with regard to maternal health outcomes among various non-English speaking communities, such as increased obstetric trauma in vaginal deliveries compared to their English-speaking counterparts [28]. Other research on language barriers that focuses on Spanish language and its impact on quality of maternal care is largely focused on prenatal care. In some ways, our study fits with this literature (perceived discrimination finding), however in other ways, our study does not (perceived medical pressure finding).

Mechanisms that describe discrimination due to language are referred to as linguistic discrimination [33]. Differences in the way one speaks can lead to judgments about a person’s socioeconomic status, class, upbringing, etc., and thus pave the way for discrimination to occur. Linguistic discrimination can have overt, subtle, and structural forms and is often tied to racial and ethnic discrimination, which may therefore describe what appeared to be a “dose–response” relationship between speaking Spanish and perceived linguistic discrimination in our results, with bilingual English/Spanish-speakers experiencing some discrimination, but overall less discrimination compared to monolingual Spanish-speakers. Discrimination due to the language someone speaks also has implications for communication issues. Prior qualitative literature in maternal care has found that discrimination due to race/ethnicity among a cohort of Black and Latina women co-occurred with negative provider communication experiences of not being listened to, and attributes of judgement, among other issues [34]. While our outcome specifically looks at perceived discrimination due to language spoken, perceived discrimination of any sort can influence the therapeutic relationship between patients and providers.

Our finding that Spanish-speaking Latina women perceived less pressure for medical interventions was unexpected based on our hypothesis. Given the vast literature on language barriers being associated with poor quality of care, we assumed this would be true for perceived pressure for an intervention during labor. The explanation may be multifactorial, and potentially related to the Latina birth paradox. This paradox demonstrates that foreign-born women of Latin American descent tend to have better birth outcomes than US-born Latina women, and the more generations that subsequently remain in the US, the more this paradox diminishes [35]. Importantly, speaking Spanish as a primary language is most likely a characteristic of those who have recently immigrated. Thus, if recently immigrated, Spanish-speaking Latina women experience low rates of poor birth outcomes, then the recommendation for medical interventions may diminish, and in turn, the perception of pressure is decreased. In other words, speaking Spanish could be acting as a proxy measure for nativity and acculturation in this case.

Looking at our two significant findings together may also illuminate another explanation. As mentioned, perceived discrimination may influence communication between patients and providers in a negative way. Miscommunication or lack of communication between a patient and provider would therefore create the inability for a provider to place pressure (and therefore for the patient to perceive pressure), providing a more nuanced conversation as to what may be occurring, and perhaps leading to our observed findings.

Additionally, Latina women perceiving less pressure for medical interventions during labor may be a result of differences in perception rather than differences in the potential actions of providers (i.e. differences in providers placing pressure) that may influence the lower perception of pressure by Spanish-speaking Latina women. Various factors may play a role in differences in perception, like culture in this case, where Latina women may not actually perceive pressure as “pressure,” but instead as normal patient care. In addition, traditional gender roles within Latinx culture, specifically Mexican culture, may also be contributing to the patient-provider relationship. *Marianismo*, is the concept of which Latina women of Mexican origin, are expected to uphold normative behaviors of femininity, submission, passivity, weakness, reservation, and virginity [36]. In this case, if providers are theoretically placing pressure for medical interventions, it is possible for the gender role of *marianismo* to show that Latina patients may revert to more passive, submissive behavior to comply with a provider’s order. However, it is important to note that culture and gender roles may influence only one aspect of perception; ultimately, Latina women are not a monolith, and as such, perception of pressure by a provider in the clinical setting must be viewed on an individual basis.

To contextualize our findings, this study is grounded in two important theoretical frameworks. Obstetric violence offers a lens through which to understand poor quality of obstetric care. Obstetric violence is a term that has gained increasing recognition in recent years, particularly within the realm of global health, as women living in under-resourced countries deal with an increase in this particular form of gender-based violence [37]. Obstetric violence has many definitions, which include some form of mistreatment, discrimination, coercion, and abuse through various means (physical, verbal, emotional, sexual, etc.) toward a laboring or birthing patient during childbirth. The definition provided by Venezuelan lawmakers and advocacy groups, where a human rights-based framework for obstetric violence has been introduced into law, provides direction toward a problem of autonomy, where obstetric violence is described as “…the appropriation of the body and reproductive processes of women by health personnel, which is expressed as dehumanized treatment, an abuse of medication, and to convert the natural processes into pathological ones, bringing with it loss of autonomy and the ability to decide freely about their bodies and sexuality, negatively impacting the quality of life of women” [38]. Not all things that are poor quality in obstetric care are considered to be obstetrically violent, however certain things like increasing cesarean rates, unnecessary episiotomies, pressuring patients into medical interventions during labor when they are not warranted, and of course, blatant physical or verbal abuse can be categorized as such [39–41]. While obstetric violence seems to be a new buzzword for the old, well-known issue of mistreatment or disrespectful childbirth care, this term brings with it the fact that individual instances of abuse in obstetrics are a part of a much broader issue of gender-based violence [38]. And unfortunately, abuse during childbirth has often been missing from the discourse surrounding gender-based violence. Although obstetric violence has gained traction internationally, obstetric violence as a term or framework in the United States has not had equal importance despite the disrespect and mistreatment in obstetric care that has been clearly described in American society historically, especially to some of the most marginalized communities including but not limited to: Black women, Spanish-speaking, immigrant women, women with substance use disorders, women with low socioeconomic status, and incarcerated women [42].

Poor quality of maternal care and lack of maternal autonomy also violates the ideology of the second framework utilized, reproductive justice. Founded by Black women, this framework defines reproductive justice as “…the human right to maintain personal bodily autonomy, have children, not have children, and parent the children we have in safe and sustainable communities” [43]. Implicit in this definition is not only the choice to birth, but also how women choose to birth, which directly relates to birthing plans, preferred modes of delivery, and quality of birthing care for all pregnant people, regardless of their ability to speak English.

Importantly, language barriers are a result of an inability to communicate in another person’s language. Often, the words used in clinical and research settings can be telling about how a particular problem is viewed (i.e. the patient as problem), and it is important to consider that when we refer to such issues as “language barriers” or “limited English proficiency,” the onus is not on the patient not being able to speak a language that is familiar to us as researchers or clinicians, but instead represents a flawed system with an inability to provide high quality, language concordant care to individuals who do not speak English, and exposes unequal power dynamics seen between English-speaking and non-English-speaking people in the US. It is important to discuss the consequences of such inequities, while also being critically aware of the implications of language differences, as well as how they are discussed and viewed [44].

Our findings should be considered in light of some limitations. While primary language was the predominant exposure variable used, primary language does not equate to a measure of English proficiency. The survey questionnaire did not ask about English proficiency (defined as being able to speak English “very well” according to the US Census Bureau) [6], nor did it ask the preferred language when communicating with providers. However, our constructed primary language variable took into account the language of the questionnaire which may suggest the language used by participants in healthcare settings. An additional limitation was the inability to determine language concordance or discordance during clinical encounters given that the survey did not ask about the language used by providers or professional medical interpretation usage. Additionally, there is also a possibility of discrimination due to place of birth, education level, and relationship status, which was not explored in the survey but could have compounded the perceived language discrimination variable. Furthermore, the survey did not ask participants to specify their country of birth, as such we could not ascertain specific nationalities or cultures of Latin America that the participants identified with. Lastly, given that this survey data included hospital births only, these findings thus, are not generalizable to non-hospital-based births.

Despite these limitations, our study is the first, to our knowledge, to examine the effect of language on patient-centered quality of care measures during birth, including discrimination due to language, perceived pressure for medical interventions, and mistreatment in a statewide representative sample. Additionally, the LTM-CA dataset recruited a large sample of Latina participants with language data available, increasing the inclusivity, generalizability, and statistical power of the study. Furthermore, this dataset included a good selection of maternal characteristics, and most of our population had complete, if not mostly complete, covariate data, allowing for a robust analysis of the data.

Future research should examine utilization of interpretation services or language data of providers, healthcare teams, as well as clinic staff to classify degree, extent, and quality of communication. Incorporating survey questions that ask specifically about English proficiency in addition to the existing language variables offered (language most often spoken at home and survey language) would also be helpful in providing context of patient encounters during birth. It is also important to expand the eligibility criteria of survey data to include participants who had home births or births in birthing centers, as this would be beneficial for comparison groups of hospital vs. non-hospital birth experiences. In addition, future research should also aim to incorporate qualitative lines of inquiry among Latina birthing people and their sense of self-efficacy, agency, and expectations about mode of birth. As well, future inquiry into perceptions of efficacy and agency in birthing and interest relating to mode of birth in people from other, Spanish-speaking countries versus people more socialized into the U.S. maternity care system would provide rich qualitative context to the results of this study.

## Conclusion

In conclusion, the results of this study demonstrated that speaking Spanish as a primary language is associated with discrimination based on language and decreased pressure for medical interventions during labor in a Latina cohort representative of the Latina birthing population in California. This suggests that Spanish language may impact some dimensions of maternal quality of care. Receiving poor quality of maternal care at the intrapartum level, may have negative health and healthcare impacts in the future. Establishing healthcare initiatives that incorporate linguistic fluency and cultural humility (the idea that one can never master competency in learning another individual’s culture, but instead one can commit to lifelong learning of other cultures in order to provide more equitable care), [45] in the labor and delivery setting is an important public health need. In addition, language concordance in conjunction with cultural, racial, and ethnic concordance between patients and providers can possibly mitigate patients’ perceptions of discrimination due to language and lead to improvement in health and quality of care outcomes [46–48]. Lastly, using an obstetric violence framework to categorize these issues in order to acknowledge obstetric violence within the broader context of gender-based violence, allows us to view poor quality of maternal birthing care as structural violence, which can then be attended to in a systemic manner [38]. Public health change that engages in these recommendations will pave the way for more equitable and reproductive justice-based birthing care.

## Data Availability

The datasets generated and analyzed during the current study are available in the University of North Carolina (UNC) at Chapel Hill Odum Institute Archive Dataverse repository, https://doi.org/10.15139/S3/3KW1DB [29]. Data included in the Odum Institute Data Archive are made publicly available on the UNC Dataverse web-accessible repository platform at no cost to depositors or users. No administrative permissions were required to access the raw data from the LTM-CA dataset used in this study.

## Abbreviations

AAPI: Asian American/Pacific Islander
aOR: Adjusted odds ratio
CI: Confidence interval
LEP: Limited English proficiency
LTM: Listening to Mothers
LTM-CA: Listening to Mothers in California
OR: Odds ratio
US: United States
WHO: World Health Organization
